# Complement factor H Val62Ile variant and risk of age-related macular degeneration: A meta-analysis

**Published:** 2013-02-13

**Authors:** Dongqing Yuan, Qin Yang, Xiaoyi Liu, Donglan Yuan, Songtao Yuan, Ping Xie, Qinghuai Liu

**Affiliations:** 1 Department of Ophthalmology, the First Affiliated Hospital of Nanjing Medical University, Nanjing, P.R. China; 2 Department of Nuclear Medicine, the First Affiliated Hospital of Nanjing Medical University, Nanjing, P.R. China

## Abstract

**Purpose:**

To evaluate the precise association of complement factor H (CFH) Val62Ile polymorphism with age-related macular degeneration (AMD) susceptibility.

**Methods:**

We performed a meta-analysis using databases including PubMed, EMBASE, and Web of Science to find relevant studies. Summary odds ratios (ORs) and 95% confidence intervals (CIs) were estimated using fixed-effect and random-effects models. The inconsistency index (I^2^) was used to assess heterogeneity. Funnel plots and Egger’s test were used to evaluate publication bias. Sensitivity analysis was also performed.

**Results:**

Fourteen studies including 4,438 patients with AMD and 6,099 controls based on the search criteria were involved in the meta-analysis. In overall populations, the pooled OR_1_ for genotype GA+GG versus homozygous genotype AA was 2.28 (95% confidence interval (CI): 1.48–3.52), the OR_2_ of heterozygous genotype GA versus AA was 1.58 (95% CI: 1.13–2.19), the OR_3_ of homozygous genotype GG versus AA was 2.90 (95% CI: 1.95–4.30), and the OR_4_ of allele G versus A was 1.77 (95% CI: 1.43–2.21). In Asian populations, our results provided substantial evidence that the Val62Ile variant was significantly associated with AMD (OR_4_=1.85, 95% CI: 1.63–2.09). However, in Caucasian populations, no significant association of Val62Ile with AMD was established in all circumstances.

**Conclusions:**

Our analysis provides substantial evidence that the Val62Ile variant is significantly associated with AMD in Asian populations. However, our results have demonstrated no link between the Val62Ile polymorphism and AMD in Caucasian populations.

## Introduction

Age-related macular degeneration (AMD), a leading cause of irreversible blindness among older individuals in developed countries, is known as a complex disease because of multiple environmental risk factors and genetic factors or the interactions among those factors [1]. The early stage of AMD is characterized by large drusen and pigmentary abnormalities in the retinal pigment epithelium. With progression to an advanced stage, AMD is manifested by geographic atrophy or the development of choroidal neovascularization and subretinal neovascular fibrous tissue (exudative or neovascular AMD) [2]. Exudative AMD includes neovascular AMD (nAMD) and serous retinal pigment epithelial detachment without choroidal neovascularization, while polypoidal choroidal vasculopathy and retinal angiomatous proliferation (RAP) have been defined as specific forms of exudative AMD [3]. Many studies have indicated that the risk of AMD increases rapidly with aging, especially in people older than 50, and the prevalence of AMD is expected to increase by 50% in the next decade [4].

Although the precise cause of AMD remains elusive, recent genetic studies have provided significant insights into the molecular basis of AMD. Some of these candidate genes such as complement factor H (CFH), high temperature required factor A1 (HTRA1), and age-related maculopathy susceptibility 2 (ARMS2) genes have been reported to increase the risk of AMD [5-7]. The Val62Ile coding variant (rs800292) in CFH on chromosome 1q32 has been extensively studied via genetic and molecular approaches, which provide strong statistical evidence for disease association and a plausible biologic context supporting this variant as an attractive candidate for a causal polymorphism leading to the development of AMD [8-10]. However, there are obvious differences in the occurrence of disease-susceptible single nucleotide polymorphisms (SNPs) between Asian and Caucasian populations [11,12]. The compelling association between Val62Ile and AMD observed in European cohorts is not as relevant to the disease risk in populations with Asian ancestry. Notably, the risk allele is less common in Asians. Its frequency is 0.614 in the HapMap database of Japanese in the Tokyo population and 0.533 in Han Chinese in the Beijing population compared with 0.808 in the CEU (Utah residents with ancestry from Northern and Western Europe) population, which makes it difficult to detect a positive signal because of insufficient statistical power. Furthermore, the population heterogeneity and bias from case-control and cohort study warrant confirmation of the association of Val62Ile with AMD across different studies in different populations. In this study, we performed a robust meta-analysis on currently available literature to assess the relationship between the Val62Ile variant and AMD.

## Methods

### Identification and eligibility of relevant studies

To search for all the studies that examined the association of the Val62Ile polymorphism with all subforms of AMD, we conducted a computerized literature search of the PubMed, EMBASE, and Web of Science databases, using the following keywords and subject terms: “macular degeneration” or “AMD,” “complement factor H,” “polymorphism,” “Val62Ile,” or other alternative names (rs800292, 184G>A, V62I, and I62V). The electronic retrieval was restricted to English literature and supplemented by the assessment of references of published studies. All related articles should have been published before December 31, 2011. Articles were included only if they met all of the following six criteria: (1) All patients had a complete ophthalmic examination, including slit-lamp biomicroscopy and fundus photography. The diagnostic criteria of AMD based on the clinical features and grading were classified using a standard grid suggested by the International Age-related Maculopathy Epidemiologic Study Group for age-related maculopathy. (2) Study design was limited to case-control study, cohort study, or population-based epidemiological survey. (3) The major study objective was to evaluate the relationship between CFH polymorphisms and all subforms of AMD. (4) The study must present available data on allele and genotype distributions for case and control subjects. The allele was G/A, and the genotypes covered GG, GA, and AA. (5) The study was written in English and published in peer-reviewed journals. (6) For repeated reports, the latest report or the report with the maximum sample numbers was selected.

### Data extraction and quality evaluation

The data extraction and quality evaluation were performed by two reviewers (DQY and QY) independently. A structured form was used to evaluate each paper according to its validity and accuracy (including the name of the first author, year of publication, ethnicity, phenotype of cases evaluated, sample size, mean age and gender ratio of study participants, methods for genotyping, and allele and genotype distributions in cases and controls). The third reviewer (XYL) would participate in a debate if the two reviewers had any disagreement on the data, and the final decision was based on the opinions of all three reviewers. The Newcastle-Ottawa Scale was also used to assess the quality of individual studies.

### Statistical analysis

Hardy–Weinberg equilibrium (HWE) was tested with a goodness-of-fit to compare the observed genotype frequencies with the expected ones among the control subjects. Software Review Manager (version 5.0, the Cochrane Collaboration, Oxford, England) was used for the meta-analysis. The following four odds ratios (ORs) and their 95% confidence intervals (95% CIs) were calculated in each study: OR_1_ for (GG+GA) versus AA, OR_2_ for GA versus AA, OR_3_ for GG versus AA, and OR_4_ for allele G versus A. The inconsistency index (I^2^) was used to test the heterogeneity. If I^2^<40%, it was considered that the heterogeneity might not be important; if I^2^ was between 30% and 60%, it may represent moderate heterogeneity; if I^2^ was between 50% and 90%, it may represent substantial heterogeneity; if I^2^ was between 75% and 100%, considerable heterogeneity exists. In the case of large heterogeneity (I^2^ >40%), random-effects models were more appropriate since they were usually more conservative. When heterogeneity was absent or moderate, random-effects and fixed-effect methods were coincided. To assess the publication bias and small-study bias, a funnel plot of the data was applied. In addition, Egger’s test was used with Stata 10.0 software (Stata Corporation, College Station, TX) to detect publication bias. We performed a sensitivity analysis by removing the unreliable study that deviated from HWE in the control group before performing the meta-analysis again.

## Results

### Selection of studies

**Figure 1:**
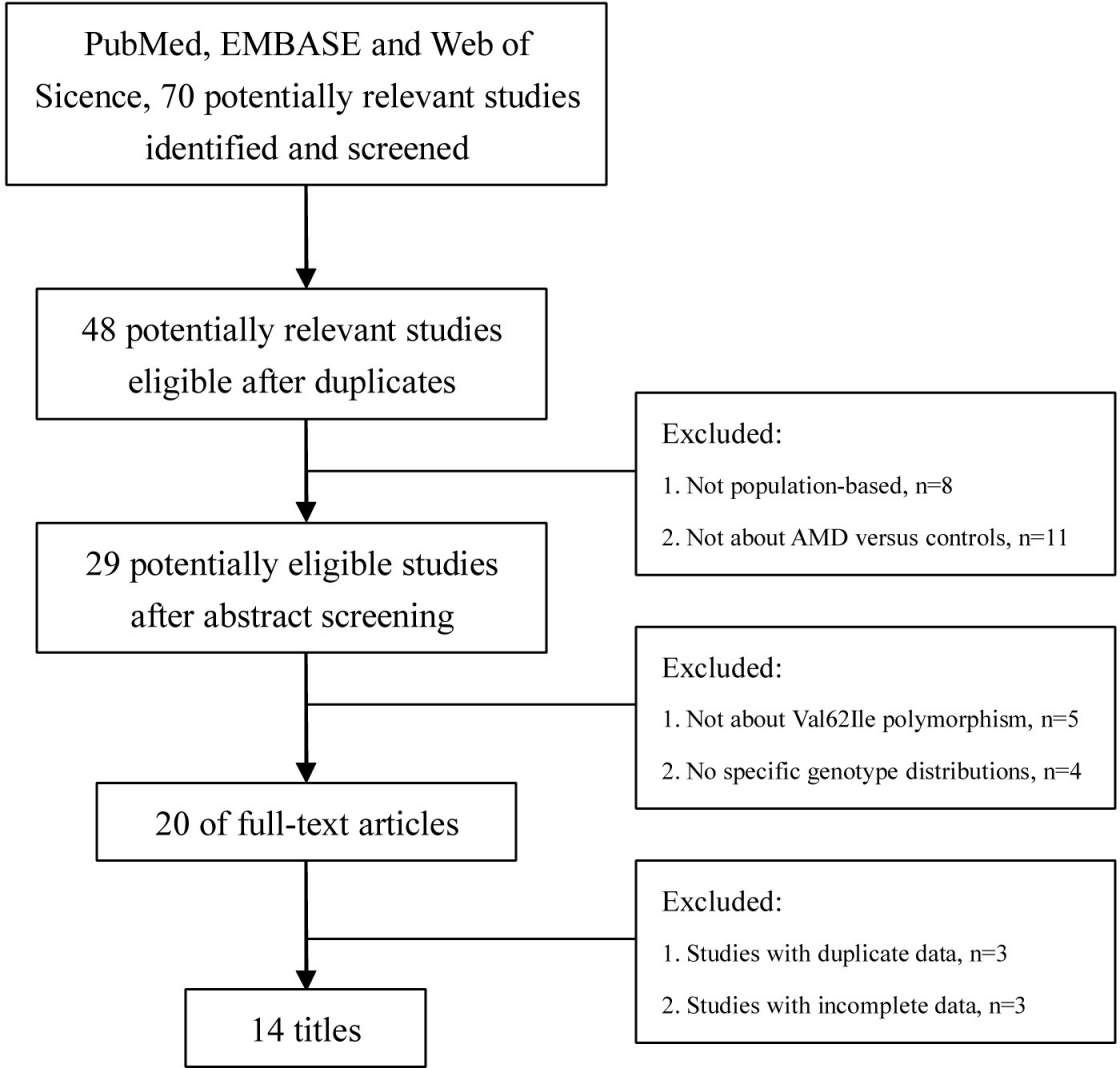
Results of the literature search strategy.

After literature search and selection applying our inclusion criteria, we identified 20 relevant articles [13-32]. Among the 20 eligible studies, two studies with duplicate data were excluded [13-15]. Moreover, three studies with incomplete data were deleted [16-18]. Finally, 14 studies containing 4,438 patients with AMD and 6,099 controls, which were intended to examine the connection between the Val62Ile polymorphism and AMD, were collected as appropriate for the meta-analysis [19-32]. Figure 1 shows the flowchart of the selection process used to identify the studies concerned. Appendix 1 lists the studies included in the meta-analysis and the summary characteristics of the study subjects. In the eligible studies, there were 10 studies of Asians and four studies of Caucasians. The average ages ranged from 63.8 to 79.5 in the case groups and 51.2 to 78.4 in the control groups. Sex ratios (male/female) in the two groups varied from 0.51 (42/83) to 3.18 (143/45) in the case groups and 0.75 (40/53) to 1.15 (722/629) in the control groups. None of the 14 studies demonstrated significant deviation from the Hardy–Weinberg equilibrium among the control subjects.

### Meta-analysis

Allele and genotype distributions for the Val62Ile variant from individual studies are shown in Table 1. The main results of this meta-analysis and the heterogeneity test are shown in Table 2. Table 3 shows the assessment of quality of all included studies with the Newcastle-Ottawa Scale.

**Table 1:** Distribution of the Val62Ile genotype for cases and controls and the allele frequencies.

Authors	Case (N)	genotype		Control (N)	genotype		^a^HWE
		GG/GA/AA	A/G		GG/GA/AA	A/G	P value
^b^Hageman et al. [19]	228	190/34/4	42/414	68	44/18/6	30/106	0.0577
^c^Hageman et al. [19]	546	395/135/16	167/925	261	148/90/23	136/386	0.0896
Chen et al. [20]	163	95/55/13	81/245	244	96/110/38	186/302	0.4883
Fuse et al. [21]	80	40/31/9	49/111	192	86/84/22	128/256	0.8286
Mori et al. [22]	188	102/71/15	101/275	139	42/73/24	121/157	0.4209
Kim et al. [23]	114	60/42/12	66/162	187	55/87/45	177/197	0.3606
Lee et al. [24]	72	41/27/4	35/109	93	36/41/16	73/113	0.4664
Xing et al. [25]	350	9/100/241	582/118	2365	154/868/1343	3554/1176	0.3901
Bergeron et al. [26]	421	324/84/13	110/732	215	123/77/15	107/323	0.5382
Goto et al. [27]	191	94/89/8	105/277	188	60/92/36	164/212	0.9447
Hayashi et al. [28]	947	538/341/68	477/1417	1338	456/649/233	1115/1561	0.9365
Hecker et al. [29]	122	91/31/0	31/213	147	85/51/11	73/221	0.392
Liu et al. [30]	222	100/92/30	152/292	235	78/119/38	195/275	0.5099
Yang et al. [31]	109	56/40/13	66/152	150	51/69/30	129/171	0.4505
Tanaka et al. [32]	685	386/263/36	335/1035	277	100/141/36	213/341	0.2091

^a^HWE=Hardy–Weinberg Equilibrium; ^b^Hageman=2005a in Iowa cohort; ^c^Hageman=2005b in Columbia cohort.

**Table 2:** Meta-analysis of the association of Val62Ile polymorphism with AMD.

Polymorphism	study	No. of studies	sample size (No.)		Test of association				Heterogeneity
			case	control	OR (95%CI)	Z	P value	Model	I2%
GG+GA ^a^vs AA	Overall	14	4438	6099	2.28 (1.48–3.52)	3.74	0.0002	^b^R	88
	Asian	10	2771	3043	2.28 (1.75–2.97)	6.13	<0.00001	R	46
	Caucasian	4	1667	3056	2.58 (0.84–7.91)	1.66	0.1	R	91
GA versus AA	Overall	14	1917	4485	1.58 (1.13–2.19)	2.71	0.007	R	75
	Asian	10	1259	1983	1.64 (1.30–2.08)	4.13	<0.00001	^c^F	27
	Caucasian	4	658	2502	1.56 (0.70–3.45)	1.09	0.28	R	79
GG versus AA	Overall	14	3003	3530	2.90 (1.95–4.30)	5.28	<0.00001	R	78
	Asian	10	1720	1578	3.18 (2.37–4.28)	7.66	<0.00001	R	52
	Caucasian	4	1283	1952	2.83 (0.76–10.62)	1.54	0.12	R	90
G versus A	Overall	14	8876	12,198	1.77 (1.43–2.21)	5.13	<0.00001	R	89
	Asian	10	5542	6086	1.85 (1.63–2.09)	9.77	<0.00001	R	46
	Caucasian	4	3334	6112	1.73 (0.91–3.30)	1.66	0.1	R	95

^a^vs: versus; ^b^R=random-effect model; ^c^F=fixed-effect model.

**Table 3:** Assessment of study quality.

Published year	study	Quality indicators from Newcastle-Ottawa Scale*								
		1	2	3	4	5A	5B	6	7	8
Case-control studies										
2005^a^	Hageman et al. [19]	Yes	Yes	No	Yes	Yes	No	No	Yes	No
2005^b^	Hageman et al. [19]	Yes	Yes	No	Yes	Yes	No	No	Yes	No
2006	Chen et al. [20]	Yes	Yes	No	Yes	No	Yes	No	Yes	No
2006	Fuse et al. [21]	Yes	Yes	No	Yes	No	No	No	Yes	No
2007	Mori et al. [22]	Yes	Yes	No	Yes	Yes	Yes	No	Yes	No
2008	Kim et al. [23]	Yes	Yes	No	Yes	Yes	Yes	No	Yes	No
2008	Lee et al. [24]	Yes	Yes	No	Yes	No	No	No	Yes	No
2009	Bergeron et al. [26]	Yes	Yes	Yes	Yes	Yes	Yes	No	Yes	No
2009	Goto et al. [27]	Yes	Yes	No	Yes	Yes	No	No	Yes	No
2010	Hayashi et al. [28]	Yes	Yes	Yes	Yes	No	No	No	Yes	No
2010	Hecker et al. [29]	Yes	Yes	No	Yes	No	No	No	Yes	No
2010	Liu et al. [30]	Yes	Yes	No	Yes	Yes	No	No	Yes	No
2010	Yang et al. [31]	Yes	Yes	No	Yes	Yes	No	No	Yes	No
2011	Tanaka et al. [32]	Yes	Yes	No	Yes	Yes	No	No	Yes	No

Cohort										
2008	Xing et al. [25]	Yes	Yes	Yes	Yes	No	No	No	Yes	No

*For case-control studies, 1, indicates cases independently validated; 2, cases are representative of population; 3, community controls; 4, controls have no history of AMD; 5A, study controls for age; 5B, study controls for additional factor(s); 6, ascertainment of exposure by blinded interview or record; 7, same method of ascertainment used for cases and controls; and 8, nonresponse rate the same for cases and controls. For cohort studies, 1 indicates exposed cohort truly representative; 2, nonexposed cohort drawn from the same community; 3, ascertainment of exposure; 4, outcome of interest not present at start; 5A, cohorts comparable on basis of age; 5B, cohorts comparable on other factor(s); 6, quality of outcome assessment; 7, follow-up long enough for outcomes to occur; and 8, complete accounting for cohorts. 2005^a^=Hageman et al.'s study in Iowa cohort; 2005^b^=Hageman et al.'s study in Columbia cohort.

### Analysis in overall populations

The association of the CFH Val62Ile polymorphism with all subforms of AMD was investigated in 14 studies with a total of 4,438 cases and 6,099 controls. We detected significant between-study heterogeneity in the comparison of (GG+GA) versus AA, GA versus AA, GG versus AA, and allele G versus A. Therefore, pooled OR_1_, OR_2_, OR_3_, and OR_4_ were all estimated based on the random-effects models. We found a significant relationship between the Val62Ile polymorphism and AMD in overall populations [(GG+GA) versus AA: OR_1_=2.28, 95% CI: 1.48–3.52; GA versus AA: OR_2_=1.58, 95% CI: 1.13–2.19; GG versus AA: OR_3_=2.90, 95% CI: 1.95–4.30; and allele G versus A: OR_4_=1.77, 95% CI: 1.43–2.21]. We also used the adjusted estimates for our analysis to minimize the bias, and the adjusted OR3 for GG versus AA were 2.945 (95% CI: 2.19–3.96) in Asian populations and 2.947 (95% CI: 0.77–11.23) in Caucasian populations (Table 4).

**Table 4:** Forest plot based on the GG versus AA group of the random-effect model with adjusted data.

Study	OR	[95% Conf. Interval]		% Weight
Asian				
Chen 2006 [20]	2.490	1.150	5.370	6.97
Fuse 2006 [21]	1.140	0.480	2.690	6.48
Goto 2009 [27]	7.050	3.070	16.200	6.64
Hayashi 2010 [28]	4.040	3.000	5.440	9.34
Kim 2008 [23]	3.957	1.835	8.535	6.98
Lee 2008 [24]	5.220	1.500	18.200	4.69
Liu 2010 [30]	1.620	0.920	2.850	8.08
Mori 2007 [22]	3.890	1.860	8.130	7.15
Tanaka 2011 [32]	2.470	1.790	3.410	9.25
Yang 2010 [31]	2.530	1.190	5.380	7.06

Sub-total				
D+L pooled OR	2.945	2.187	3.965	72.64

Caucasian				
Bergeron 2009 [26]	3.700	1.600	8.300	6.69
Hageman 2005a [19]	6.480	1.750	23.930	4.46
Hageman 2005b [19]	3.840	1.970	7.460	7.54
Hecker 2010 [29]	24.610	1.430	424.130	1.46
Xing 2008 [25]	0.310	0.150	0.640	7.21

Sub-total				
D+L pooled OR	2.947	0.774	11.226	27.36

Overall				
D+L pooled OR	2.788	1.922	4.046	100.00

OR=odds ratio in random-effect model; 95% CI=95% confidence interval.

### Analysis in Asian populations

The meta-analysis included 10 studies (2,771 cases and 3,043 controls) in Asian populations. We detected significant between-study heterogeneity in all other comparisons, and the random-effects model was used in that situation except GA versus AA. The heterogeneity with the I^2^ test showed no statistical significance in GA versus AA models, and the fixed-effect model was used to evaluate the association of Val62Ile with AMD. Our analysis provides substantial evidence that the Val62Ile variant is significantly associated with AMD in Asian populations [(GG+GA) versus AA: OR_1_=2.28, 95% CI: 1.75–2.97; GA versus AA: OR_2_=1.64, 95% CI: 1.30–2.08; GG versus AA: OR_3_=3.18, 95% CI: 2.37–4.28; allele G versus A: OR_4_=1.85 and 95% CI: 1.63–2.09].

### Analysis in Caucasian populations

The meta-analysis included four studies (1,667 cases and 3,056 controls) in Caucasian populations. The I^2^ test of heterogeneity was significant in all the comparisons of OR_1_, OR_2_, OR_3_, and OR_4_. Therefore, the random-effects model was used in all comparisons. No significant association of Val62Ile with AMD was established in four contrasts in Caucasian populations [(GG+GA) versus AA: OR_1_=2.58, 95% CI: 0.84–7.91; GA versus AA: OR_2_=1.56, 95% CI: 0.70–3.45; GG versus AA: OR_3_=2.83, 95% CI: 0.76–10.62; and allele G versus A: OR_4_=1.73, 95% CI: 0.91–3.30].

### Evaluation of publication bias

The shapes of the funnel plots were used to evaluate evidence of obvious asymmetry (funnel plots not shown). Meanwhile, we assessed funnel plot asymmetry with Egger’s linear regression test. The intercept provided a measure of asymmetry, and the larger the deviation from zero, the more pronounced the asymmetry. The results of Egger’s linear regression test are shown in Appendix 2. The results of the funnel plots are shown in Appendix 3, Appendix 4, and Appendix 5. There was no publication bias for all comparisons in Asian populations and Caucasian populations. However, in the contrasts of the GG+GA versus AA model and the GA versus AA model in the overall populations, the shapes of the funnel plots were slightly asymmetric. Then, the Egger’s test results indicated significant publication bias in the two comparisons.

### Sensitivity analysis

In our analysis, no study deviated from HWE in the control groups. However, in the Xing et al. study the genotype distribution was completely different from the others. Therefore, we performed sensitivity analysis by removing that study. In the overall populations, the results of the sensitivity analysis were as follows: [(GG+GA) versus AA: OR_1_=2.44, 95% CI: 1.93–3.08; GA versus AA: OR_2_=1.69, 95% CI: 1.37–2.08; GG versus AA: OR_3_=3.35, 95% CI: 2.62–4.29; and allele G versus A: OR_4_=1.93 and 95% CI: 1.75–2.13]. In the Caucasian populations, the results of the sensitivity analysis were significantly different [(GG+GA) versus AA: OR_1_=3.22, 95% CI: 2.02–5.11; GA versus AA: OR_2_=1.97, 95% CI: 1.14–3.39; GG versus AA: OR_3_=3.96, 95% CI: 2.49–6.29; and allele G versus A: OR_4_=2.15 and 95% CI: 1.82–2.55].

## Discussion

In the present study, our meta-analysis focused on the relationship between the Val62Ile polymorphism and AMD risk in different populations. In the overall populations, we found a significant association between the Val62Ile variant and AMD. In Asian populations, the results of the meta-analysis suggested that the G allele was significantly associated with AMD risk. However, in Caucasian populations, based on four case-control studies, no significant link between the Val62Ile polymorphism and AMD was detected under all genetic models. However, in the sensitivity analysis after the Xing et al. study was removed, the Val62Ile polymorphism was significantly associated with AMD risk in Caucasian populations.

Variants in the CFH gene, such as Y402H (Tyr402His) and Val62Ile, have been shown to be strongly associated with AMD in different ethnic groups; however, there are obvious differences in the occurrence of disease-susceptible SNPs between Asian and Caucasian populations [11,12,20,25]. Although the causative nature of the Val62Ile variant has not been fully proven, several lines of evidence provide significant insight into the mechanist basis for the association between the risk allele defined by this variant and AMD. Notably, Tortajada et al. [33] reported that the Val62Ile substitution in short consensus repeat (SCR) 1 of CFH increases its affinity for C3b; thus, when compared to CFH-Val62, CFH-Ile62 competes more efficiently with complement factor B (CFB) for C3b binding in proconvertase formation and acquires enhanced cofactor activity for the complement factor I (CFI) mediated cleavage of C3b proteolysis; however, its decay accelerating activity is not altered. These findings show that CFH-Ile62 is a better alternative pathway convertase inhibitor and provides an explanation for the association of the CFH-Ile62 variant with protection in three distinct disorders linked by alternative pathway dysregulation. The fact that the Val62Ile substitution affects binding to C3b but not decay-accelerating activity suggests that different regions in CFH may be involved in binding C3b/cofactor activity and in decay-accelerating activity.

Heterogeneity is a potential problem when interpreting results of all meta-analyses [34]. In our analysis, significant heterogeneity between studies may exist in overall comparisons in each genetic model except the GA versus AA model in Asian populations. The observed heterogeneity could be attributable to differences in several factors such as ethnic variations, environmental factors, and methodological factors in the design and conduct of the studies. Among these risk factors, ethnic variations could play a crucial role. After subgroup analysis by race, the heterogeneity was effectively decreased in Asian populations, but no significant change was found in Caucasian populations. To minimize the bias, we have tried to do our analysis as follows: First, we used the Newcastle-Ottawa Scale to assess the quality of individual studies [34], and the results were shown in the revised manuscript. Second, we performed a publication bias analysis for all four comparisons. Third, we recalculated all the statistics, and the adjusted estimates were used in our general analysis: the adjusted OR of (GG versus AA) in overall populations indicated that patients with homozygote of risk allele might be at twofold risk for AMD. Due to the small number of studies in our research, the random-effects model was more acceptable to improve the accuracy of our conclusions. In the contrasts of the GG+GA versus AA model and the GA versus AA model in the overall populations, the shapes of the funnel plots and the Egger’s test results showed significant publication bias; however, the results of Egger’s test in Asian and Caucasian populations did not show significant publication bias in the two contrasts. Race might be the crucial factor for the different results of AMD risk. Xing et al.’s [25] regression coefficient estimates suggest the minor allele G of Val62Ile is deleterious to the filtering capacity of the kidney, but protective against AMD susceptibility, which is completely different from other studies. They concluded that the Val62Ile variant may not be the major genetic determinant underlying the connection between the renal and ocular traits, and the allelic architecture of causal variants for these two diseases may be fairly complex. That might explain the high publication bias among the overall populations. Meanwhile, the results of the sensitivity analysis in Caucasian populations also demonstrated that the Val62Ile variant was significantly associated with AMD by removing that study. Furthermore, in Chen et al.’s study [20], three other alleles, T, G, and C, respectively, for SNPs in the promoter (rs3753394), exon 2 (rs800292), and intron 15 (rs1329428) located in a common haplotype TGTC (with an estimated haplotype frequency of 56%), and the loci rs800292 and rs3753394, which were in high linkerd dimorphisms (LD) with rs1329428, were significantly associated with exudative AMD adjusted for age, gender, and smoking. Mori et al. [22] also reported that the V62I variant was in moderately high LD with rs14100996 and rs2274700. The gene-gene interaction was tested to be one of the risk factors for AMD.

The present meta-analysis has several limitations. First, we restricted our search to studies published in English; thus, we may have missed articles published in other languages. Second, a more precise analysis should be conducted if we increase the samples and estimate them based on the adjusted analysis. Third, because of the complex nature of AMD, it is unlikely that an SNP in a single gene would be obviously associated with an increase in AMD risk, without consideration of other polymorphic susceptible genes. Except CFH Val62Ile variants, other complement factors gene polymorphisms such as Y402H, C3 and CFB, which have been studied extensively in different populations [35-38], need to be studied in this population to understand the impact of these variations on the onset and progression of AMD. Finally, since the number of studies included in each comparison of our research was limited, especially in Caucasian populations, the conclusions remain to be confirmed by further studies.

In conclusion, our analysis provides substantial evidence that the CFH Val62Ile variant is significantly associated with AMD in Asians populations. However, our results failed to demonstrate the link between the Val62Ile polymorphism and AMD in Caucasian populations. Further prospective research, with more participants and fully confounding risk factors considered, is warranted to examine the possible effects of this variation on AMD.

## Appendix 1.

General characteristics of the included studies. To access the data, click or select the words “Appendix 1.”

## Appendix 2.

Egger’s linear regression test to measure the funnel plot asymmetric. To access the data, click or select the words “Appendix 2.” Egger’s linear regression test to measure the funnel plot asymmetric. The results indicated significant publication bias in two comparisons of “GG+GA vs AA” and “GA vs AA” in overall populations.

## Appendix 3.

To access the data, click or select the words “Appendix 3.” The results of the funnel plots in all comparisons in overall populations. A was in comparison of “GG+GA vs AA”; B was in comparison of “GA vs AA”; C was in comparison “GG vs AA”; and D was in comparison “G vs A”.

## Appendix 4.

To access the data, click or select the words “Appendix 4.” The results of the funnel plots in all comparisons in Asian populations. A was in comparison of “GG+GA vs AA”; B was in comparison of “GA vs AA”; C was in comparison “GG vs AA”; and D was in comparison “G vs A”.

## Appendix 5.

To access the data, click or select the words “Appendix 5.” The results of the funnel plots in all comparisons in Caucasion populations. A was in comparison of “GG+GA vs AA”; B was in comparison of “GA vs AA”; C was in comparison “GG vs AA”; and D was in comparison “G vs A”.
